# Facilitating Role of the 3D Viewing System in Tilted Microscope Positions for Cataract Surgery in Patients Unable to Lie Flat

**DOI:** 10.3390/jcm11071865

**Published:** 2022-03-28

**Authors:** Otman Sandali, Rachid Tahiri Joutei Hassani, Ashraf Armia Balamoun, Mohamed El Sanharawi, Vincent Borderie

**Affiliations:** 1 Centre Hospitalier National d’Ophtalmologie des XV-XX, Pierre & Marie Curie University Paris 06, ResearchTeam 968, Institut de laVision, 75571 Paris, France; borderie@quinze-vingts.fr; 2 Service de Chirurgie Ambulatoire, Hôpital Guillaume-de-Varye, 18230 Bourges, France; 3 Service de Chirurgie Ambulatoire, Centre Hospitalier de Granville, 50400 Granville, France; tjhr78@hotmail.com; 4 Watany Eye Hospital WEH, Cairo 11775, Egypt; ashrafarmia@gmail.com; 5 Watany Research and Development Centre, Cairo 11775, Egypt; 6 Ashraf Armia Eye Clinic, Giza 12655, Egypt; 7 Service D’ophtalmologie, Centre Hospitalier de Châteaudun, 28200 Chateaudun, France; mohamed.elsanharawi@gmail.com

**Keywords:** cataract surgery, tilted microscope positions, 3D viewing system, patients unable to lie flat

## Abstract

Purpose: To assess the utility of the 3D viewing system in tilted microscope positions for the performance of cataract surgery in challenging positions, for patients with difficulty remaining supine. Methods: Prospective, single-center, single-surgeon, consecutive case series of patients undergoing surgery in an inclined position. Results: 21 eyes of 15 patients who had undergone surgery at inclined positions at angles of 20° to 80°, with a mean angle of 47.62°. Surgeon comfort was considered to be globally good. The surgeon rated red reflex perception and the impression of depth as good and stable in all cases. The operating time was slightly longer for patients inclined at angles of more than 50°. On the first day after surgery, BSCVA was 20/25 or better in all cases. No ocular complications occurred in any of the interventions. Conclusions: Due to the ocular-free design of the 3D system, the surgical procedure and the positioning of the surgeon remained almost identical to that for patients undergoing surgery in a supine position, maintaining the safety of the standard surgical approach.

## 1. Introduction

Ocular surgery is usually performed in patients lying in a supine position with the surgical microscope perpendicular to the surgical plane.

However, this usual position may not be possible if the patient cannot remain in a supine position due to medical conditions. Rotating the optical axis of the microscope perpendicular to the eye is one possible solution for such procedures in patients undergoing surgery in an inclined position [1,2,3,4]. However, it is very challenging to perform surgeries when the microscope rotation exceeds 30° in practice, because a greater rotation is incompatible with the posture of the surgeon, who needs to be able to look through the microscope oculars during surgery.

The three-dimensional (3D) digital visualization system was recently evaluated and shown to be safe for ocular surgery [5,6,7]. The ocular-free design of the 3D system makes it possible for the surgeon to adopt a much more ergonomic posture during surgery and may release the limitations on the axis of the microscope rotation.

In this study, we report the utility of the 3D viewing system in tilted microscope positions for cataract surgery in challenging positions in 15 consecutive patients unable to remain in a supine position.

## 2. Surgical Technique

This report included consecutive patients undergoing cataract surgery in an inclined position for medical reasons, at Guillaume de Varye Hospital (Bourges, France) between January 2021 and November 2021. These patients were either unable to remain in a supine position or found this position very uncomfortable. The study was approved by the ethics committee of our institution and was performed in accordance with the Declaration of Helsinki.

All the interventions were performed by the same experienced surgeon (O.S.) with the Constellation^®^ (Alcon Surgical, Ft. Worth, TX, USA) surgical system and the 3D digital visualization system (NGENUITY^®^, Alcon, Fort Worth, TX, USA), connected to a (Lumera 700 Carl Zeiss Meditec, Jena, Germany) microscope.

All the operations were performed under topical anesthesia. The patients were lying in a standard reclining cataract surgical chair, the back of which was reclined to a position in which the patient felt comfortable, to ensure that the surgical conditions were good.

**Figure 1:**
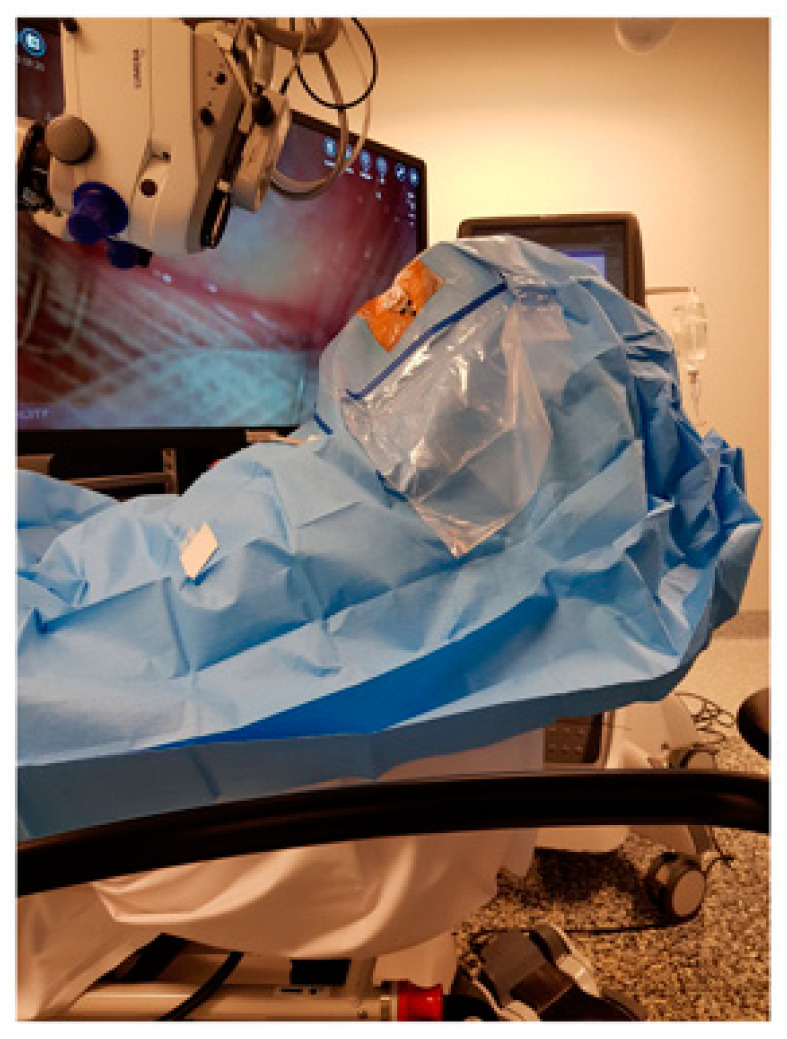
Patient suffering from orthopnea undergoing cataract surgery in an inclined position. The microscope was tilted perpendicular to the surgical plane.

The surgical chair was lowered as much as possible, to ensure that the patient’s eye was located at about the generator cassette level. Depending on the angle at which the patient was tilted, the microscope was tilted such that it was parallel to the eye and its optical axis was perpendicular to the surgical plane, providing good visualization (Figure 1).

**Figure 2:**
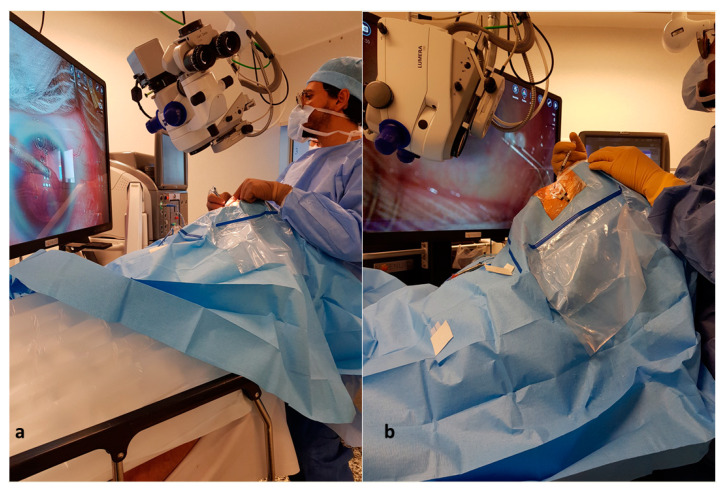
Surgeon positioning behind patients who underwent cataract surgery in inclined positions ((**a**): patient inclined in a 30° position. (**b**): patient inclined in a 65° position).

The surgeon sat, as usual, behind the patients and a 2.2 mm principal corneal incision was made in the superotemporal quadrant for right eyes, and in the superonasal quadrant for left eyes, avoiding the eyebrow (Figure 2). The nucleus was emulsified by the divide and conquer technique (Video S1 Supplementary Material).

Pre-operative cataract grading was assessed according to a simplified nuclear classification score based on the posterior nuclear color appearance [8].

Red reflex perception, the impression of depth, the operating time, the need for corneal suture, operative complications, and surgeon comfort (scale: 1–3; 1: comfortable, 2: mild discomfort, 3: uncomfortable) were assessed with a questionnaire.

## 3. Results

In total, 21 eyes from 15 patients were enrolled in the study (Table 1). Most of these patients (9/15) had degenerative spinal disorders.

Patients were inclined at angles of 20° to 80°, with a mean angle of 47.62°. The surgeon considered red reflex perception and the impression of depth to be good and stable in all cases, as in surgeries performed with patients lying flat. No ocular complications occurred in any of the interventions. None of the patients required corneal suture. Operating time was slightly longer for the patients inclined at angles of more than 50° (*p* < 0.01).

Surgeon comfort was rated “1” (comfortable) in all cases in which the patient was inclined at less than 60° and “2” (mild discomfort) for patients inclined at angles exceeding 60°.

On the first day after surgery, BSCVA was 20/25 or better in all cases.

## 4. Discussion

In this series, we evaluated the facilitating role of the ocular-free design of the 3D visualization system to the performance of surgeries in unusual challenging positions using the microscope rotation, in cataract surgery on patients unable to remain supine.

Microscope tilting is used in other indications in patients undergoing operations in a supine position. Indeed, by displacing the angle of view, this technique allows the visualization of the trabecular meshwork in stent implantation or the extension of the peripheral retinal view in retinal surgery [9,10].

At high angles of standard microscope rotation, the surgeon becomes very uncomfortable and must change his posture and modify the surgical approach, sometimes even modifying the location of the incision, which may increase the risk of operative complications. In a series of 32 eyes, Richard et al. reported the results for a face-to-face upright seated position for cataract surgery in patients who cannot lie supine, with the surgeon either seated or standing, and facing the patient [2]. Inferior, temporal or inferotemporal corneal incisions were made. Capsular rupture occurred in two cases, with nucleus drop. The authors considered this surgical positioning technically challenging and recommended its use only by experienced surgeons. Muraine et al. recently reported a series of four eyes in which face-to-face phacoemulsification was performed, with a slit lamp and the surgeon sitting facing the patient and performing a temporal incision [11].

In our series of 21 eyes, due to the ocular-free design of the 3D system, the surgical procedure and the positioning of the surgeon for patients undergoing cataract surgery in an inclined position remained almost identical to that for patients undergoing surgery in a supine position. The safety of the standard surgical approach was, therefore, maintained.

Within the eye, the quality of visualization, the impression of depth and red reflex perception were considered to be very good and similar to those in standard operating conditions.

A fast and good visual recovery was recorded in all cases on the first day after surgery. The good visualization conditions and the perceived depth of field may have ensured the safety of intraocular maneuvers, accounting for this result.

In conclusion, we reported here the facilitating role of the ocular-free design of the 3D system for the performance of ocular surgery in unusual challenging positions in patients who are unable to lie flat. This system makes it possible to maintain the usual position and the safety of the standard surgical approach in such challenging conditions.

## Figures and Tables

**Table 1:** Clinical characteristics and outcomes of patients who underwent cataract surgery in inclined positions.

Patient	Side	Age (Years)	Comorbidity	Cataract Grading	Preoperative BCVA	Patient Inclination (Degrees)	Surgery Duration (Minutes)	Impression of Depth	Red Reflex Perception	Surgeon Comfort *	1 Day after Surgery BCVA
1	R	91	Orthopnea	3	20/70	50	16	Good	Good	1	20/20
2	R	82	Positional Vertigo	2	20/50	40	12	Good	Good	1	20/20
2	L	82	Positional Vertigo	2	20/50	40	11	Good	Good	1	20/20
3	L	75	Back pain	4	20/100	30	10	Good	Good	1	20/25
4	R	54	Neck pain	2	20/40	45	10	Good	Good	1	20/15
4	L	54	Neck pain	2	20/50	45	12	Good	Good	1	20/15
5	R	69	Orthopnea	3	20/70	70	15	Good	Good	2	20/25
5	L	69	Orthopnea	2	20/50	70	16	Good	Good	2	20/25
6	R	72	Orthopnea	2	20/40	80	17	Good	Good	2	20/20
7	R	78	Back pain	2	20/50	60	17	Good	Good	2	20/25
8	R	80	Neck pain	2	20/50	20	9	Good	Good	1	20/25
8	L	80	Neck pain	2	20/40	20	9	Good	Good	1	20/20
9	R	59	Back pain	4	20/100	35	11	Good	Good	1	20/25
10	R	74	Orthopnea	4	20/100	65	18	Good	Good	2	20/25
11	R	83	Neck pain	3	20/70	35	10	Good	Good	1	20/25
11	L	83	Neck pain	3	20/70	35	9	Good	Good	1	20/25
12	R	75	Neck pain	2	20/50	40	12	Good	Good	1	20/15
12	L	75	Neck pain	2	20/50	40	12	Good	Good	1	20/15
13	R	81	Orthopnea	2	20/30	65	15	Good	Good	2	20/15
14	L	68	Back pain	2	20/50	55	15	Good	Good	1	20/20
15	L	77	Positional Vertigo	3	20/70	60	16	Good	Good	2	20/25

BCVA: Snellen best corrected visual acuity. * Surgeon comfort was assessed with a questionnaire (scale: 1–3; 1: comfortable, 2: mild discomfort, 3: uncomfortable).

## Data Availability

The data that support the findings of this study are available from the corresponding author, O.S., upon request.

## Supplementary Materials

The following supporting information can be downloaded at: https://www.mdpi.com/article/10.3390/jcm11071865/s1, Video S1: Video highlighting the surgeon’s installation and the surgical procedure in a patient who underwent cataract surgery in an inclined position.

## References

[1] Ang G.S., Ong J.M., Eke T. (2006). Face-to-face seated positioning for phacoemulsification in patients unable to lie flat for cataract surgery. Am. J. Ophthalmol..

[2] Lee R.M.H., Jehle T., Eke T. (2011). Face-to-face upright seated positioning for cataract surgery in patients who cannot lie flat. J. Cataract Refract. Surg..

[3] Sohail T., Pajaujis M., Crawford S.E., Chan J.W., Eke T. (2018). Face-to-face upright seated positioning for cataract surgery in patients unable to lie flat: Case series of 240 consecutive phacoemulsifications. J. Cataract Refract. Surg..

[4] Pajaujis M., Injarie A., Eke T. (2013). Extreme face-to-face positioning for cataract surgery with patient seated upright in motorized wheelchair. J. Cataract Refract. Surg..

[5] Weinstock R.J., Diakonis V.F., Schwartz A.J., Weinstock A.J. (2019). Heads-Up Cataract Surgery: Complication Rates, Surgical Duration, and Comparison with Traditional Microscopes. J. Refract. Surg..

[6] Freeman W.R., Chen K.C., Ho J., Chao D.L., Ferreyra H.A., Tripathi A.B., Nudleman E., Bartsch D.U. (2019). Resolution, Depth of Field, and Physician Satisfaction during Digitally Assisted Vitreoretinal Surgery. Retina.

[7] Sandali O., El Sanharawi M., Tahiri J.H.R., Roux H., Bouheraoua N., Borderie V. (2021). Early corneal pachymetry maps after cataract surgery and influence of 3D digital visualization system in minimizing corneal oedema. Acta Ophtalmol..

[8] (2020). Mandelblum J, Fischer N, Achiron A, Goldberg M, Tuuminen R, Zunz E, Spierer O: A Simple Pre-Operative Nuclear Classification Score (SPONCS) for Grading Cataract Hardness in Clinical Studies. J. Clin. Med..

[9] Ohno H. (2019). Utility of Three-Dimensional Heads-Up Surgery in Cataract And Minimally Invasive Glaucoma Surgeries. Clin. Ophthalmol..

[10] Sandali O., Tahiri J.H.R., Duliere C., El Sanharawi M., Borderie V. (2022). Use of a 3D viewing system and microscope tilting to extend the peripheral retinal view. RETINA.

[11] Muraine M., Boutillier G., Toubeau D., Gueudry J. (2019). Face-to-face phacoemulsification using a slitlamp in patients who are unable to lie flat. J. Cataract Refract. Surg..

